# Apigetrin inhibits adipogenesis in 3T3-L1 cells by downregulating PPARγ and CEBP-α

**DOI:** 10.1186/s12944-018-0738-0

**Published:** 2018-04-25

**Authors:** Fatma Hadrich, Sami Sayadi

**Affiliations:** Environmental Bioprocesses Laboratory, AUF Regional Excellence Pole (AUF-PER-LBP), Sfax Biotechnology Center, P.O. Box 1177, 3038 Sfax, Tunisia

**Keywords:** Apigetrin, Adipogenesis, Cell cycle, ROS, TNF-α, IL-6

## Abstract

**Background:**

Apigetrin, a flavonoid found in many plant leaves and seeds, has been known to possess antimutagenic, anti-cancer, antioxidant and anti-inflammatory properties. Here, we are investigating the effect of the apigetrin on adipocytes differentiation in 3T3-L1 adipocytes, and elucidating the mechanism of its action.

**Methods:**

Lipids accumulation was measured by Oil Red O staining and cell cycle was analyzed by flow cytometry. The antioxidant effect of apigetrin was evaluated against hydrogen peroxide. The expression of various genes, involved in adipogenesis and inflammation, was studied by real-time PCR.

**Results:**

Our results showed that apigterin treatment inhibited significantly lipid accumulation without effect on cell viability at 100 μM, and it exerted the anti-adipogenic effect during the early stages of differentiation. Flow cytometry analysis showed that apigenin-7-O-glucoside (Ap7G) inhibited cell proliferation during mitotic clonal expansion and caused cell cycle delay. Quantitative PCR analysis revealed that the mRNA levels of C/EBP-α, PPAR-γ, SREBP-1c and FAS were suppressed after apigetrin treatment at 100 μM. Moreover, the mRNA level of pro-inflammatory genes (TNF-α and IL-6) were suppressed after apigterin treatment, at high concentration preadipocyte cells.

**Conclusion:**

Taken together, these results indicated that apigenin-7-O-glucoside inhibits adipogenesis of 3T3-L1 preadipocytes at early stage of adipogenesis.

## Background

The prevalence of obesity has rapidly increased in the past several decades and became a global health problem [1]. Obesity is often characterized by physical inactivity and consumption of a high-fat/cal diet and associated with lifestyle-related diseases, such as hyperlipidaemia, hypertension [2], arteriosclerosis, type 2 diabetes mellitus, cancer, respiratory complications, and osteoarthritis [3].

At the cellular level, obesity is defined by an increase in adipose tissue mass, which is the result of an enlargement in fat cells and/or an increase in their number [4]. Adipogenesis is the process of preadipocytes differentiation into adipocytes. Several transcription factors are regulated including the CCAAT/enhancer-binding protein (C/EBP) gene family and peroxisome proliferator activated receptor-γ (PPARγ) [5]. These transcription factors are also involved in the sequential expression of adipocyte specific proteins [6, 7]. Therefore, inhibition of adipogenesis is critical for achieving an anti-obesity effect and the search for agents that could control this process has been extensively undertaken [8].

Natural products derived from plants are receiving a lot of attention as treatment options and as chemopreventive agents. Flavonoids, are naturally occurring polyphenolic compounds abundant in food plants, have attracted significant public attention due to their various biological activities [9]. These products possess many biological and pharmacological activities owing to their potential anticancer, anti-inflammatory, antioxidant, and antimicrobial properties, as well as important nutritional supplements to the human diet [10, 11]. Their anti-obesity effect can act through various mechanisms by preventing weight gain and this may be an excellent alternative strategy for developing safe anti-obesity drugs. Among flavonoids compounds that have been tested for potential anti-obesity, we cited the quercitin, rutin, naringenin, luteolin and resveratrol [12, 13].

Apigenin is found abundantly in vegetables and fruits and it has a variety of physiological properties such as antioxidant, anti-inflammatory [14]. Added to its low toxicity, this flavonoid has been shown to have selective effects in inhibiting cell growth and inducing apoptosis in cancer cells [15]. Apigenin has been used as a dietary supplement and was reported to exert anti-obesity effect in 3T3-L1 cells through activation of AMPK [16].

The pure form of apigenin is unstable but in natural form in food, it is present mostly as glucoside conjugate, which is called apigetrin. The conjugated form could be an important determinant for its bioavaiblity and absorption. Previous reports have investigated the pharmacological effects of the apigetrin including anti-inflammatory, antioxidant and anti-diabetic effects. However, its anti-adipogenic effect remains unclear. Therefore, the objective of this study was to investigate the effect of the apigetrin on mouse preadipocyte differentiation and to elucidate the mechanism underlying its effect, with a particular focus on the expression of molecules involved in adipogenesis.

## Materials and methods

### Reagents

3T3-L1 cells were provided from the Health Science Research Resources Bank (HSRRB, Osaka, Japan). Apigetrin was purchased from Extrasynthese Company (Genay, France). Dulbecco’s modified Eagle’s medium (DMEM high-glucose), Dexamethasone, 3- iso-butyl-1- methylxanthine, and Insulin were purchased from Sigma-Aldrich (Missouri, USA). MTT was provided from Gibco, DCFH-DA, was purchased from Sigma Aldrich.

Primers against PPARγ, C/EBPα, FAS, SREBP-1c, TNF-α and IL-6 were purchased from Santa cruz Biotechnology (Beverly, MA, USA).

### Cell culture

3T3-L1 cells were cultured in DMEM medium containing 10% FBS at 37 °C and 5% CO2. Cells were plated at a density of 2 × 10^5^ cells. After reaching the confluence, adipocyte differentiation was initiated using the same medium containing 10 mg/L insulin, 0.5 mmol/L isobutylmethylxanthine, and 1 μmol/L dexamethasone for 2 days. The medium was then replaced with DMEM, containing 5 mg/L insulin for more 2 days, and then changed to fresh medium every 2 days. Apigetrin was diluted in DMSO, the final quantity of the solvent was 0.1% for control and treated cells for all experiment.

### Oil red O staining and quantification

After differentiation, 3T3-L1 cells were washed twice with phosphate buffered saline (PBS, pH 7.4), fixed with lipid droplet assay fixative solution (Cayman kit) for 1 h, and then stained with Oil red O solution (in 60% isopropanol) at room temperature for 10 min. After washing, cells were checked and pictures were taken using a microscope (BioZero BZ-8000; Keyence, Osaka, Japan). Moreover, the dye was extracted with dye extraction solution and the absorbance (OD. 420 nm) was measured by a Spectra Max microplate reader (Spectra Max 190; Molecular Devices Corporation, CA, USA).

### MTT assay

3T3-L1 cells were harvested in 96-well plate. After reaching the confluence, the culture medium was replaced by 100 μL containing Apigetrin (0–200 μmol/L) and the cells were incubated for further 48 h. The culture medium was removed and replaced by 100 μL of fresh culture medium containing 10% of sterile filtered MTT (Sigma-Aldrich). After 6 h, the insoluble formazan crystals were dissolved in 100 μL/well SDS and the absorbance was measured at 570 nm. The inhibition (%) was expressed as the percentage of viable cell compared to control.

### Trypan blue assay

3T3-L1 cells were seeded for 2 days after confluence in the presence of apigetrin. Then, cells viability was quantified by Trypan Blue assay. After washing twice with PBS, cells were trypsinized and immediately stained with 0.5% trypan blue dye (Trypan Blue, Sigma-Aldrich) for 3 min. Cells were observed under an optical microscope, and the viability was calculated as the percentage ratio of the number of unstained cells relative to the total cells counted.

### Measurement of intracellular ROS level

Intracellular reactive oxygen species (ROS) level was measured using a fluorescent dye, DCFH-DA according to [17]. Differentiated cells (Day 7) were cultured in DMEM containing, 400 μM H_2_O_2_ for 4 h and then with 30 μM DCFH-DA for 30 min. Then, fluorescence intensity (excitation/emission 485/528 nm) was measured using a Varioscan HT plate reader. The values were calculated as a percentage (%) of control.

### Flow cytometry analysis

Post-confluent 3T3-L1 cells were treated with differentiation media in the absence or presence of several doses of apigetrin for 24 h. Both detached and adherent cells were collected by trypsinization and washed with phosphate buffered saline (PBS). Cells were fixed with 70% ethanol at 4 °C for overnight. After removing of ethanol, cells were stained with propidium iodide (Sigma-Aldrich) for 30 min in the obscurity. Fluorscencent cells analysis was carried out by using Guava EasyCyte (Guava Technologies, Hayward, CA, USA).

### Gene expression analysis

Total RNA was extracted from 3T3-L1 cells by acid-GTC-phenol method [18]. After DNase I (Takara Bio, Otsu, Shiga, Japan) treatment and RNA repurification, the cDNA was synthesized using M-MLV Reserse Transcriptase (Takara), and subjected for PCR. Quantitative PCR analysis was carried out using SYBR Premix Ex Taq (Takara). Each cDNA was amplified (95 °C for 5 s, 60 °C for 30 s, 72 °C for 30 s, for 40 cycles) using specific primers (Table 1).

**Table 1:** Primers for RT-PCR. PCR was performed using the primers indicated as below under optimal amplification condition (95 °C for 5 min; 22–35 cycles of 95 °C for 30 s, 58 °C for 30 s, 72 °C for 30 s; 72 °C for 7 min) for each gene

Name	Forward	Revoerse
GAPDH	5′-TGGTGAAGGTCGGTGTGAACGG-3′	5′-TGCCGTTGAATTTGCCGTGAGT-3′
PPARγ	5′-AAACTCTGGGAGATTCTCCT-3′	5′-TGGCATCTCTGTGTCAAC-3′
C/EBPα	5′-GCCAAACTGAGACTCTTC-3′	5′-TGGCATCTCTGTGTCAAC-3′
SREBP-1c	5′-GCTTAGCCTCTACACCAACTGGC-3′	5′-ACAGACTGGTACGGGCCACAAG-3′
FAS	5′-TGGAGCCTGTGTAGCCTTCGAG-3′	5′-ACAGCCTGGGGTCATCTTTGCC-3′
IL-6	5′- GGTGACAACCACGGCCTTCCC -3’	5′- GCCACTCCTTCTGTGACTCCAGC -3’
TNF-α	5′- AAATGGGCTCCCTCTCATCAGTTC-3’	5′- TCTGCTTGGTGGTTTGCTACGAC-3’

### Statistical analysis

The results of three experiments were pooled and expressed as mean ± standard deviation (SD). Data were subjected to a one-way analysis of variance (ANOVA) using Graph Pad Prism 6.0 followed by Tukey’s multiple comparison test at *p* < 0.05.

## Results

### Apigetrin inhibits MDI-induced adipogenesis of 3T3-L1 preadipocytes

**Fig. 1:**
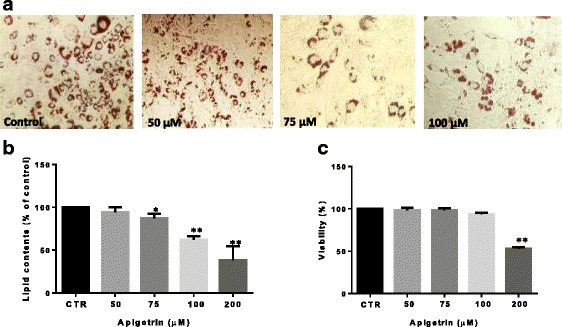
**a** Effect of apigetrin on adipocyte differentiation. Adipogenesis was induced in the presence or absence of several dose of apigetrin for 8 days. Then, intracellular lipid accumulation was stained by oil red O solution. The intracellular lipid accumulation was expressed as a percentage of control values. **b** Cytotoxicity of apigetrin on 3T3-L1 preadipocytes. The cells were cultured until confluence and then, cells were treated with Ap7G at indicated concentrations for 24 h. **c** Cell viability was measured by MTT assay. The viability was expressed as a percentage of control values. Data were presented as means ± S.D. (*n* = 3). The asterisks (**) indicate a significant difference between control group and MDI-treated group(*p* < 0.01)

To investigate the anti-adipogenic effect of apigetrin, 3T3-L1 preadipocytes were induced to differentiate with MDI in the presence or absence of apigetrin, and cells were stained with oil red O solution. Our results showed that apigetrin dose-dependently inhibits adipogenesis of 3T3-L1 preadipocytes (Fig. 1a and b). Intracellular lipid accumulation was reduced after apigetrin treatment by 5%, 37% and 62% at 50, 100 and 200 μM, respectively compared to untreated group (Fig. 1b). To check whether this anti-adipogenic effect of apigetrin is due to its cytotoxicity, the effect of apigetrin on cell viability was measured by MTT assay. Our results showed that apigetrin does not exhibit cytotoxicity up to 100 μM (Fig. 1b) suggesting that anti-adipogenic of apigetrin is not from cytotoxicity, but through regulation of other molecular mechanism. Together, these results suggest that apigetrin has anti-adipogenic effect in 3T3-L1 preadipocytes.

### Apigetrin inhibits early stage of differentiation

To investigate the mechanism of anti-adipogenic effect of apigetrin during early phase of differentiation, 3T3-L1 cells were treated in the presence of different concentrations of apigetrin over 0–2 days (early stage), 2–4 days (middle stage), 6–8 days (late stage).

**Fig. 2:**
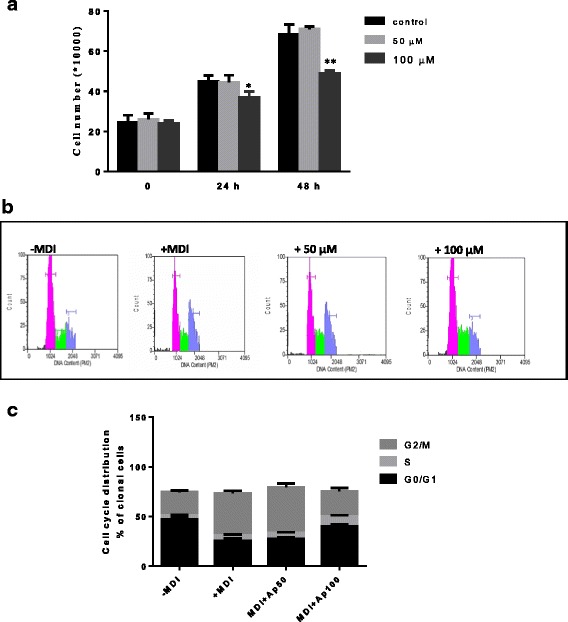
**a** Effect of Ap7G on MDI induced cell number increase and cell cycle progression (**b** and **c**). Differentiation of 3T3-L1 preadipocytes was initiated in the presence of Ap7G (0, 50, 100 μmol/L). After 24 h and 48 h, the cells were trypsinized and counted. Final concentration of DMSO was 0.1%. Change of cell cycle was analyzed by flow cytometry (**b**) and plotted on graph (**c**). The flow cytometry was performed 3 independent times. Data were presented as means ± S.D. (*n* = 3). The asterisks (*) and (**) indicate a significant difference between control group and MDI-treated group (*p* < 0.05) and (*p* < 0.01), respectively

As shown in Fig. 2a**,** apigetrin exhibited anti-adipogenic effects essentially in the early stage. During the middle and late stages, its effect was very low, with no significant difference seen between the control and the treated cells.

### Effect of apigetrin on the clonal expansion and cell cycle progression of 3T3-L1 cells during the early stage of differentiation

As described above, Ap7G displayed its main effect during the early stage of differentiation. We thus anticipated that this compound would affect the preadipocyte proliferation step.

Trypan blue assay result showed that following 24 h and 48 h exposure, apigetrin at 100 μM decreased DMI-induced clonal expansion and the cell number remained lower in the treated culture (Fig. 2b). Next, cell cycle profile was examined by FACS analysis. Our results showed that apigetrin treatment caused a significant delay in the progression of the cell cycle and increased G0/G1 and S population in a dose-dependent manner (Fig. 2c) without any effect in the detection of dividing cells (G2M).

### qRT-PCR analysis

**Fig. 3:**
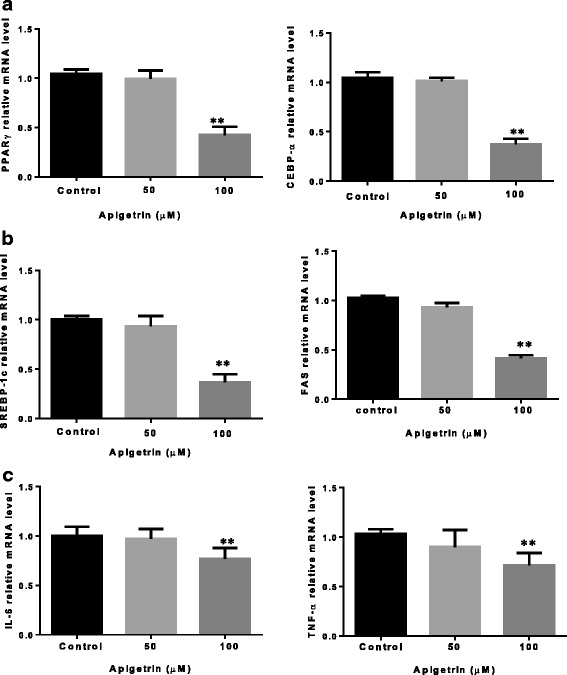
**a** and **b** Effect of apigetrin on gene expression of PPARγ, CEBP-α, SREBP-1c and FAS. **c** Effect of Ap7G on TNF-α and IL-6 gene expression 3T3-L1 cells were cultured 8 days after initiation of differentiation. Cells were treated with 0–100 μmol/L of Ap7G or for 8 days at 37 °C in a humidified 5% CO2 incubator. The relative expression level of PPARγ, CEBP-α, SREBP-1c, FAS, TNF-α and IL-6 was quantified by qRT-PCR. Final concentration of ethanol was 0.1%. Data were presented as means ± S.D. (*n* = 3). The asterisks (**) indicate a significant difference between control group and MDI-treated group (*p* < 0.01)

Several transcription factors, such as the C/EBP and PPAR families, are sequentially and cooperatively expressed during differentiation. In this study, we evaluated whether the decreases in intracellular lipid contents were associated with lower levels of PPAR-γ and C/EBP-α, expressed in the early stage of adipogenesis. As shown in Fig. 3, Ap7G (100 μM) markedly suppressed MDI-induced up-regulation of PPAR-γ and C/EBP-α with no significant effect at 50 μM (Fig. 3a). Expression of both adipogenic marker proteins was not detected after 2 days of MDI treatment, representing the early stage of adipogenesis. Similarly, this compound was able to decrease the mRNA level of SREBP-1c and FAS (Fig. 3b). Moreover, Ap7G treated 3T3-L1 cells decreased the level of the pro-inflammatory genes especially TNF-α and IL-6 (Fig. 3c).

### Effect of Apigetrin on ROS production

**Fig. 4:**
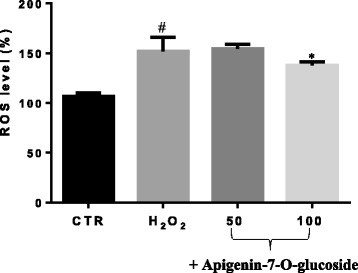
Effects of Apigetrin on ROS levels in 3T3-L1 adipocytes. Cells were treated with different concentration of apigetrin for 24 h and then treated with H_2_O_2_ (0.4 mM) for 30 min. ROS levels were assessed by fluorescence intensity using DCFH-DA. All values are presented as means ± S.D. (*n* = 3). * Statistically significant compared to H_2_O_2_ alone (*P* < 0.05). ^#^statistically significant compared to control

To investigate the capacity of the apigenin-7-O-glucoside to reduce H_2_O_2_ induced ROS production, we use the fluorescence probe DCFH-DA. Our results showed that the adipocytes cells exposed to H_2_O_2_ showed an increase in the intracellular level of ROS compared to the untreated cells used as a control (Fig. 4). However, treated cells with apigetrin reduced significantly (*p* < 0.05) the ROS level of about 21% at 100 μM, respectively.

## Discussion

Adipocyte differentiation and fat deposition are correlated to the emergence and development of obesity. Hence, many researchers advise that adipose tissue should become a main drug target for obesity and lipid metabolism dysregulation. The prevention and treatment of obesity with drugs is usually associated with the appearance of adverse reactions and drug dependence. For this reason, recent studies have been focusing on natural substances without side effects for the prevention or therapy of obesity [19]. Flavonoids are abandant in fruits, seeds and vegetables, and they have protective effects against various diseases [20]. Flavonoids showed different biological activities depending on their chemical structures; for example, the number and location of hydroxyl groups, their conjugation pattern and the position of sugar in their structure [21].

In this study, we revealed for the first time the effect of apigetrin in 3T3-L1 preadipocyte model. Previously, Rauter et al., [22] have reported the anti-hyperglcemic of Ap7G on streptozotocin-Induced diabetic rats. Our results showed that apigetrin suppressed lipid accumulation in a dose-dependent manner. In fact, Ap7G inhibited triglyceride accumulation by around 5% and 37% at 50 and 100 μM, respectively without any toxicity at high concentration. Our results are in concomitance with the report of Chao et al., 2013 who have demonstrated that flavonoids bounded to sugar present lower toxicity in either in vitro and in vivo models [23]. Although data shortage concerning the anti-obesity effect of Ap7G, Li et al., 2005 have revealed the anti-adipogenic effect of genistin, analogue of apigetrin, in bone morrow stem cells (MSC) at 10 μM [24]. Moreover, many studies showed that flavonol glycoside, isolated from plants, exhibited a strong inhibition against lipids accumulation, in adipocytes and hepatocytes cells [25, 26].

MCE is an important step in the adipocyte differentiation process. Thus, blocking DNA replication by various means prevents differentiation.

Time-course analysis of the effect of apigetrin showed that this compound was able to exhibit its strongest effects during the early stages of differentiation, which runs in parallel with clonal expansion. Interestingly, Ap7G decreased DMI-induced clonal expansion of 3T3-L1 cells and delayed the cell cycle progression in a dose-dependent manner. These data are consistent with several previous studies, which showed that arresting or delaying the cell cycle of 3T3-L1 cells by phenolic compounds during the first 2 days of differentiation, decreased cell number and inhibited the differentiation rate of adipocytes. In this context, Hsu et al., [27] have reported that genistein, resveratrol and kaempferol, inhibits adipocyte differentiation during mitotic clonal. Ono et al., [16] have demonstrated that apigenin was able to decrease the accumulation of intracellular lipids in 3T3-L1 cells and regulated the expression of adipogenic genes during the first days.

PPARγ is involved in the regulation of fat cell genes adipocytes and is the key nuclear transcription factor that regulate different cellular functions, such as adipocyte lipogenesis, fatty acid transport, and energy metabolism. Furthermore, C/EBPα was found to be a regulator of cell differentiation. It is expressed at the adipogenic initiation stage and synergistically triggers adipocyte -specific gene expression with PPAR-γ after the growth arrest stage [28]. Our findings showed that Ap7G inhibited the gene expression of PPARγ and C/EBPα, which lead to a reduction in fat deposition. Previous studies have confirmed that, apigenin suppressed adipocyte differentiation by reducing PPARγ, C/EBPα mRNA levels and activating AMPK-activated protein kinase [16, 29]. Similarly, Park et al., [30] have reported that luteolin-7-O-glucoside inhibited the expression of adipogenic gene and down-regulated the expression of proteins involved in adipocytes differentiation.

Previous reports have shown that FAS can be a potential drug target because its inhibition can reduce food intake and obesity in mice [31]. The SREBP-1c appears to be involved and early expressed in adipocyte differentiation. It is the main regulator of FAS expression in liver and adipose tissues [32]. As shown in our results, apigetrin treatment inhibited significantly FAS mRNA expression at 100 μM. Our findings suggest that blocking SREBP-1c activation by Ap7G resulted in a significant reduction in FAS expression. These results are in accordance with the research of Morikawa et al., who have demonstrated that flavonoids glycoside inhibits the expression of the transcription factors involved in the adipocyte differentiation [25].

The obesity is usually associated with increased mitochondrial ROS production, causing the oxidized lipids, synthesis of faulty proteins, and mtDNA mutations, which are related to decreased metabolic activity, mitochondrial dysfunction, and cellular insulin sensitivity [33]. Moreover, it has been established that high ROS production leads to a dysregulation of adipokine secretion and contribute to the production of pro-inflammatory cytokines such as: tumor necrosis factor- α (TNF-α), interleukin-6 (IL-6), monocyte chemoattractant protein-1 (MCP-1) and the reduction on adiponectin secretion [34]. In the present work, we found that apigetrin decreased the intracellular ROS level induced by the hydrogen peroxide and decreased the mRNA gene levels of TNF-α and IL-6 at high concentration. Similarly, treated mature adipocytes with apigetrin decreased TNF-α secretion (data not shown). Our findings are in accordance with those reported by Francisco et al., [35], who showed that flavonoids, such as quercitin, genistein and luteolin glycosides showed potential anti-inflammatory agents, due to their significant inhibitory effects on ROS and NO levels produced by LPS-induced inflammation in mouse macrophage cells. Similarly, Miguel et al., [36] have demonstrated that pretreatment of animals with 50 mg/kg of apigetrin inhibited the LPS induced the production of TNF- α and IL-6. In the same context, Hamalainen et al., have reported that genistin, was less effective on the iNOS inhibition and NO production in murine J774 macrophage [37]. Previous studies have shown that the O-glycosylation of flavonoids decreased the antioxidant activity of isoflavonoids due to the presence of the sugar that may attenuate the absorption of the flavonoid aglycone and thus reduced its activity. However, no clear structural activity relationship depending on the positions or types of sugar substitution was found in the anti-inflammatory activity for flavonoids glycosides.

## Conclusion

The present study reveals that apigenin-7-O-glucoside can serve as a potential candidate for the management of obesity. It exerted inhibitory effects on 3T3-L1 preadipocytes differentiation and caused cell delay. As shown in molecular analysis, apigetrin treatment decreased the mRNA levels of PPARγ, CEBP-α, SREBP-1c and FAS genes involved in the adipocyte differentiation. This compound was also able to reduce the pro-inflammtory gene expression Further studies are being carried out to validate its effects on other biological process related to adipocyte biology.

## Abbreviations

C/EBPα: CCAAT enhancer binding protein alpha
DEX: Dexamethasone
DMEM: Dulbecco’s modified Eagle’s medium
FAS: Fatty acid synthase
IBMX: 3-isobutyl-1-methylxanthine
IL-6: Interleukine-6
PPARγ: Peroxisome proliferator-activated receptor-gamma
SREBP-1c: Sterol regulatory element-binding proteins
TNF-α: Tumor necrosis factor
